# Can Ocular Optical Biomarkers Predict Response to Contact-Lens Myopia Control? A Critical Review of Current Evidence

**DOI:** 10.3390/diagnostics16213479

**Published:** 2026-10-24

**Authors:** Ahmed Abass

**Affiliations:** Department of Materials, Design and Manufacturing Engineering, University of Liverpool, Liverpool L69 3GH, UK; a.abass@liverpool.ac.uk

**Keywords:** myopia control, contact lenses, ocular optical biomarker, prediction, axial elongation, relative peripheral refraction, higher-order aberrations, retinal image quality, orthokeratology

## Abstract

**Background:** Contact-lens interventions can slow, but do not arrest, axial elongation in children, and individual response varies substantially. The review examined whether early optical measurements of the lens–eye system can provide clinically useful prediction of later axial growth. **Methods:** A critical narrative review of PubMed-indexed literature was updated through October 2026. Evidence from dual-focus, centre-distance multifocal and extended-depth-of-focus soft contact lenses and orthokeratology was appraised according to temporal ordering, repeatability, independent prediction, external validation and potential clinical utility. Spectacle-based myopia-control designs were outside the primary scope. **Results:** Relative peripheral refraction (RPR), pupil-weighted retinal image-quality metrics and selected higher-order aberration (HOA) measures are the principal optical candidates. In soft-lens wear, early wide-field RPR change has shown moderate discrimination of later axial growth. In orthokeratology, the most developed HOA evidence concerns total HOA root-mean-square (RMS), primary spherical aberration and coma; change in total anterior corneal HOA RMS has shown the strongest single-study discrimination. Accommodation, lens centration, pupil size and wear time are better treated as modifiers of optical exposure than as ocular optical biomarkers. Positive findings remain largely small, single-centre, post hoc or design-specific, and no candidate has undergone adequate independent external validation. **Conclusions:** Current evidence supports candidate-predictive signals rather than clinically validated biomarkers. No optical measure has demonstrated sufficient incremental value beyond established clinical factors or shown that biomarker-guided management improves outcomes. Modality-specific or multivariable optical signatures remain more plausible than a universal scalar marker.

## 1. Introduction

Current myopia-control interventions slow rather than arrest progression, and treated eyes generally continue to elongate. The ability of contact lenses to reduce the rate of childhood myopia progression is supported by a substantial clinical evidence base. Randomised trials have demonstrated reduced axial elongation with dual-focus, high-add centre-distance multifocal, extended-depth-of-focus (EDOF) and orthokeratology strategies, although the magnitude of benefit varies between designs and between children [1,2,3,4,5]. The more difficult question is why apparently similar treatment produces a strong response in some children and continued rapid elongation in others.

Age and baseline progression provide important clinical context for treatment response [1], but they do not describe the optical state produced at the retina during wear. For daytime contact lenses, the intended optical profile is present only while the lens is worn, so verified wear time should be recorded as part of treatment exposure. The prescribed lens is only the starting point. Pupil size, lens centration, accommodation, ocular aberrations, treatment-zone geometry and viewing behaviour can all alter the retinal image formed by a given design [6,7,8].

A useful optical predictor should therefore describe the lens–eye system rather than the product label alone. In this review, the term “ocular optical biomarker” refers to a measurable optical characteristic of the eye or lens–eye system that can be obtained before treatment or early during wear and tested against subsequent axial elongation. Candidate measures include peripheral refraction, ocular or corneal wavefront metrics and derived pupil-weighted retinal image-quality metrics. Accommodation, pupil diameter, lens centration and treatment-zone geometry can alter the optical state at the retina and are therefore treated primarily as delivery modifiers or surrogates rather than biomarkers in their own right. Retinal electrophysiology is considered separately as a biological response. This terminology keeps ocular optical characteristics distinct from biological retinal responses.

The available literature already contains several signals that meet part of this definition. Early changes in wide-field relative peripheral refraction have shown temporally ordered associations with later axial growth in soft contact-lens studies, while accommodative behaviour has emerged as a potentially informative response modifier; larger randomised data provide important counterevidence against simple peripheral defocus or pupil size as stand-alone predictors [9,10,11]. In orthokeratology, treatment-induced higher-order aberrations and spatial peripheral-refraction measures have been studied more extensively in relation to later growth [12,13,14,15,16,17]. Overall, predictive information appears to be present, but no candidate has yet demonstrated the reproducibility, independence and external validation required for routine clinical use. The review therefore examines which optical measures have moved beyond mechanistic plausibility towards prediction, where the evidence remains inconsistent, and whether modality-specific or multivariable signatures may ultimately be more useful than a single universal marker.

## 2. Scope and Critical-Review Approach

A critical narrative approach was used to examine contact-lens-based interventions for progressive childhood and adolescent myopia. The targeted PubMed-indexed search was updated through 11 October 2026 and supplemented by reference tracking from major randomised trials, systematic reviews, meta-analyses and International Myopia Institute reports. Search concepts included myopia control, contact lens, dual focus, centre-distance multifocal, extended depth of focus, orthokeratology, axial elongation, relative peripheral refraction, retinal image quality, higher-order aberration, spherical aberration, coma, pupil size, centration, accommodation, treatment zone, back optic-zone diameter, prediction, responder, biomarker, receiver-operating characteristic and predictive performance. Randomised and prospective controlled studies were prioritised for treatment efficacy, while prospective cohorts, post hoc trial analyses and optical studies were included when they examined whether an early measurable variable was associated with or predicted later axial growth.

The purpose was not to produce a pooled treatment-effect estimate. Instead, the evidence was appraised according to whether it addressed efficacy, association or prediction. An optical variable may change after treatment and may even correlate with axial elongation without being useful for prediction. Greater weight was therefore given to temporal ordering, measurement repeatability, independence from recognised confounders, prospective performance and external validation. Spectacle-based interventions, including aspheric/lenslet, bifocal and progressive designs, were deliberately excluded because the review focuses on optical signals generated by contact-lens wear or by the corneal reshaping produced by orthokeratology. The two contact-lens modalities were also treated separately where appropriate: daytime soft lenses deliver their optical profile only while worn, whereas orthokeratology is worn overnight and creates a changing daytime corneal optical state after lens removal. Broader efficacy evidence for soft multifocal lenses and orthokeratology has already been synthesised in recent reviews and meta-analyses [18,19,20,21].

## 3. What Would Count as an Ocular Optical Biomarker?

### 3.1. Association, Mechanism and Prediction Are Different Questions

The term biomarker can easily become broader than the evidence allows. Three levels are useful in this context. A mechanistic marker changes in a manner consistent with a proposed biological pathway. An associated marker is statistically related to axial elongation. A predictive biomarker goes further: when measured at baseline or early after treatment, it improves prediction of subsequent axial growth in a new patient or dataset. Only the last of these has a realistic prospect of supporting treatment selection or early adaptation.

This distinction is important because several design variables are repeatedly associated with efficacy without themselves constituting ocular optical biomarkers. Smaller orthokeratology treatment zones, for example, are associated with better average axial control [22,23,24,25], but back optic zone diameter is a lens-design parameter rather than a measured ocular optical characteristic. Its relevance is mediated through the optical state created on eye, including peripheral power distribution, pupil overlap and induced aberrations. The same caution applies to labelled add power: a group-level dose-response can be present even when the optical state produced at the retina in an individual child remains uncertain [3,6].

### 3.2. Practical Criteria for a Useful Biomarker

A clinically useful ocular optical biomarker would ideally satisfy several practical requirements. The measurement should be repeatable under defined conditions, available before treatment or early enough to influence management, and predictive of later axial elongation after accounting for age and baseline progression. Its interpretation should also remain reasonably stable across instruments and viewing conditions, and its predictive value should be reproduced outside the cohort in which it was discovered. A marker measured only after the treatment response has already become obvious may be mechanistically informative, but it has limited value as an early clinical predictor. These appraisal criteria are summarised in Table 1.

## 4. Candidate Optical Biomarkers

### 4.1. Relative Peripheral Refraction and Peripheral Myopic Defocus

Relative peripheral refraction (RPR) remains one of the most widely studied optical candidates because peripheral retinal defocus has long been implicated in eye-growth regulation. Both myopia-control soft lenses and orthokeratology commonly shift peripheral refraction in a myopic direction, and RPR can be measured clinically [1,26,27]. The difficulty is that its relationship with axial response varies between lens designs, retinal locations and analytical approaches. In the BLINK study, several peripheral-defocus measures were associated with slower axial growth, but the associations were markedly attenuated when randomised treatment assignment was included in the model; photopic and mesopic pupil size also did not modify the high-add multifocal treatment effect [11]. These findings make a single peripheral-defocus value or pupil diameter an unlikely stand-alone explanation of soft-lens response.

A more encouraging soft-lens signal was reported in a 2025 prospective MiSight cohort using wide-field multispectral refraction topography. Changes in total and regional RPR at three months were negatively correlated with subsequent annual axial growth, with the strongest correlations around r = −0.43. Receiver-operating-characteristic analyses produced areas under the curve of 0.70–0.76 for selected RPR changes [9]. The study was small and had no parallel control group, but the early timing and moderate discrimination place RPR closer to a predictive marker than a purely mechanistic observation. Prospective orthokeratology studies have also linked later axial elongation to RPR features measured in particular annuli, quadrants and defocus-ring geometries [12,13,14].

The apparent difference between the BLINK and MiSight findings is more useful when treated as a methodological clue than as a simple contradiction. BLINK examined whether measured peripheral defocus independently explained a randomised treatment effect across lens groups, whereas the MiSight study examined whether an early change in a wide-field RPR map distinguished later, faster and slower growth within a treated cohort [9,11]. The lenses, spatial sampling and statistical questions were different. The reported correlations of approximately r = −0.43 are statistically meaningful but explain less than one fifth of the variance, so they should not be interpreted as sufficient for individual clinical prediction. Instrument sampling also matters. The Grand Seiko WAM-5500, widely used for peripheral refraction, has an effective measurement-beam diameter of about 2.46 mm at the anterior cornea [28]. When contact-lens power changes rapidly across small zones, a finite sampling aperture can average across distinct optical regions. Peripheral-refraction studies should therefore report the instrument aperture, pupil state and sampling geometry explicitly, particularly when measurements are obtained under conditions that differ from habitual viewing. The present evidence supports region-specific or map-based RPR as a promising candidate, while showing that its predictive value is likely to depend on how and where it is measured. No pre-specified RPR threshold has yet been replicated across centres or lens designs.

### 4.2. Higher-Order Aberrations: Total HOA RMS, Spherical Aberration and Coma

Higher-order aberrations (HOAs) comprise multiple Zernike components and should not be treated as one interchangeable optical quantity. The evidence considered in this review is concentrated on total HOA root-mean-square (RMS), spherical-aberration metrics and coma. In the longitudinal study by Lau et al. [16], total ocular HOA RMS combined third- to sixth-order terms; spherical-aberration RMS combined Z4^0 and Z6^0; and comatic RMS combined Z3^−1, Z3^1, Z5^−1 and Z5^1. Higher total HOA RMS and spherical-aberration RMS, and specifically more positive primary spherical aberration Z4^0, were associated with slower axial elongation. Other individual higher-order terms may change with orthokeratology, but the reviewed literature does not provide comparable longitudinal or predictive validation for trefoil, tetrafoil/quadrafoil, secondary astigmatism or other individual Zernike components [15,16].

The strongest recent predictive evidence comes from a 2025 prospective orthokeratology cohort [17]. The study analysed anterior corneal total HOA RMS, primary spherical aberration Z4^0, vertical coma Z3^−1 and horizontal coma Z3^1 over the central 6-mm cornea. Changes in total HOA RMS, peripheral defocus and horizontal coma were independently related to axial elongation. Change in total anterior corneal HOA RMS (ΔHOA RMS) produced an area under the receiver-operating-characteristic curve of 0.803, with a proposed cut-off of approximately 0.834 micrometres for distinguishing axial elongation of ≤0.1 from >0.1 mm/year; change in horizontal coma showed a separate AUC of 0.756 [17]. The 0.834 micrometre threshold therefore refers to change in total anterior corneal HOA RMS, not to spherical aberration or coma.

The available evidence therefore supports a hierarchy rather than a generic HOA label. Total HOA RMS currently has the strongest prospective discrimination, primary spherical aberration has the clearest longitudinal association, and horizontal coma has emerging predictive value in orthokeratology [16,17]. The other individual HOA terms considered in broader optical studies should not be assumed to have equivalent predictive value. Longitudinal evidence linking an early HOA metric to individual response with soft multifocal or EDOF lenses remains limited. In the MiSight Assessment Study Spain, baseline optical and binocular variables did not produce a convincing optical predictor of progression; time outdoors emerged more clearly in the small predictive analysis [29].

### 4.3. Treatment-Zone Position, Size and Pupil Overlap

Treatment-zone geometry has one of the most reproducible relationships with orthokeratology efficacy. Randomised and controlled studies show that smaller treatment zones or modified back optic--zone diameters can reduce axial elongation [22,23,24,25]. Observational work has also associated treatment-zone decentration with axial elongation, although this does not justify deliberate decentration in clinical fitting [30]. These findings are important for lens engineering, but treatment-zone size and position are better interpreted as delivery surrogates than as ocular optical biomarkers themselves.

The retinal image produced by orthokeratology depends on how the reshaped cornea interacts with pupil size and the ocular wavefront. Smaller or decentred zones alter the distribution of non-central power and aberrations across the pupil, so treatment-zone geometry, RPR and HOAs often change together [22,23,24,25,30]. Centration is also relevant to soft lenses: modern soft lenses can be systematically displaced relative to the pupil centre even when they appear well centred relative to the cornea [31]. Because pupil diameter and viewing conditions also change the fraction of a zonal design sampled by the eye [6,7,8], task-specific or repeated on-eye measurements may be more informative than a single centration value. Geometry may therefore be useful for optimisation even when it does not identify the specific retinal-image feature responsible for the response.

### 4.4. Pupil-Weighted Retinal-Image Quality

Retinal image-quality metrics are attractive because they combine several optical effects into a single representation of the image reaching the retina. Point-spread function, modulation transfer, visual Strehl and through-focus image-quality measures can incorporate pupil size, defocus and aberrations rather than treating them as independent variables. Optical characterisation has shown that commercially available myopia-control lenses with similar indications can produce materially different retinal image quality [7,32], while synthetic accommodative modelling demonstrates distinct through-focus image-quality patterns for dual-focus and EDOF designs [8].

The missing step is longitudinal validation. Most image-quality studies describe optical performance or visual consequences but do not obtain an early pupil-weighted metric and test it prospectively against axial elongation months later. Because retinal image quality integrates the whole wavefront, it may ultimately prove more informative than any single RPR or HOA term. At present, however, it is better regarded as a promising measurement framework than as a validated predictor.

### 4.5. Accommodation and Dynamic Optical Exposure

Accommodation changes the optical state at the retina during near viewing and is best regarded as a physiological modifier of optical exposure rather than an ocular optical biomarker itself. A post hoc analysis of a one-year trial involving 109 children measured accommodative response after one week of wear with either a single-vision lens or a positive-spherical-aberration myopia-control soft lens. Within the treatment group, greater accommodative response was associated with less axial elongation at one year (regression coefficient −0.59 mm, *p* < 0.0001), whereas the corresponding association was not significant in controls [10]. The early timing is favourable for prediction, although the finding is design-specific, post hoc and has not been independently replicated across contemporary dual-focus or EDOF lenses.

More recent work reinforces the dynamic nature of the optical state produced at the retina. During 60 min of sustained electronic-device viewing, children wearing dual-focus MiSight lenses showed changes in accommodative behaviour and increasing hyperopic defocus while the treatment zones continued to introduce myopic defocus [33]. These measurements did not predict later axial growth, but they illustrate why a single static distance measurement may not represent habitual optical exposure at the retina. Accommodation, pupil size, centration and wear time should therefore be recorded as modifiers of optical exposure and, where justified, tested as predictive covariates. BLINK provides no evidence that pupil size alone should determine selection for high-add multifocal treatment [11].

### 4.6. Contrast-Based and Electrophysiological Signals

**Figure 1:**
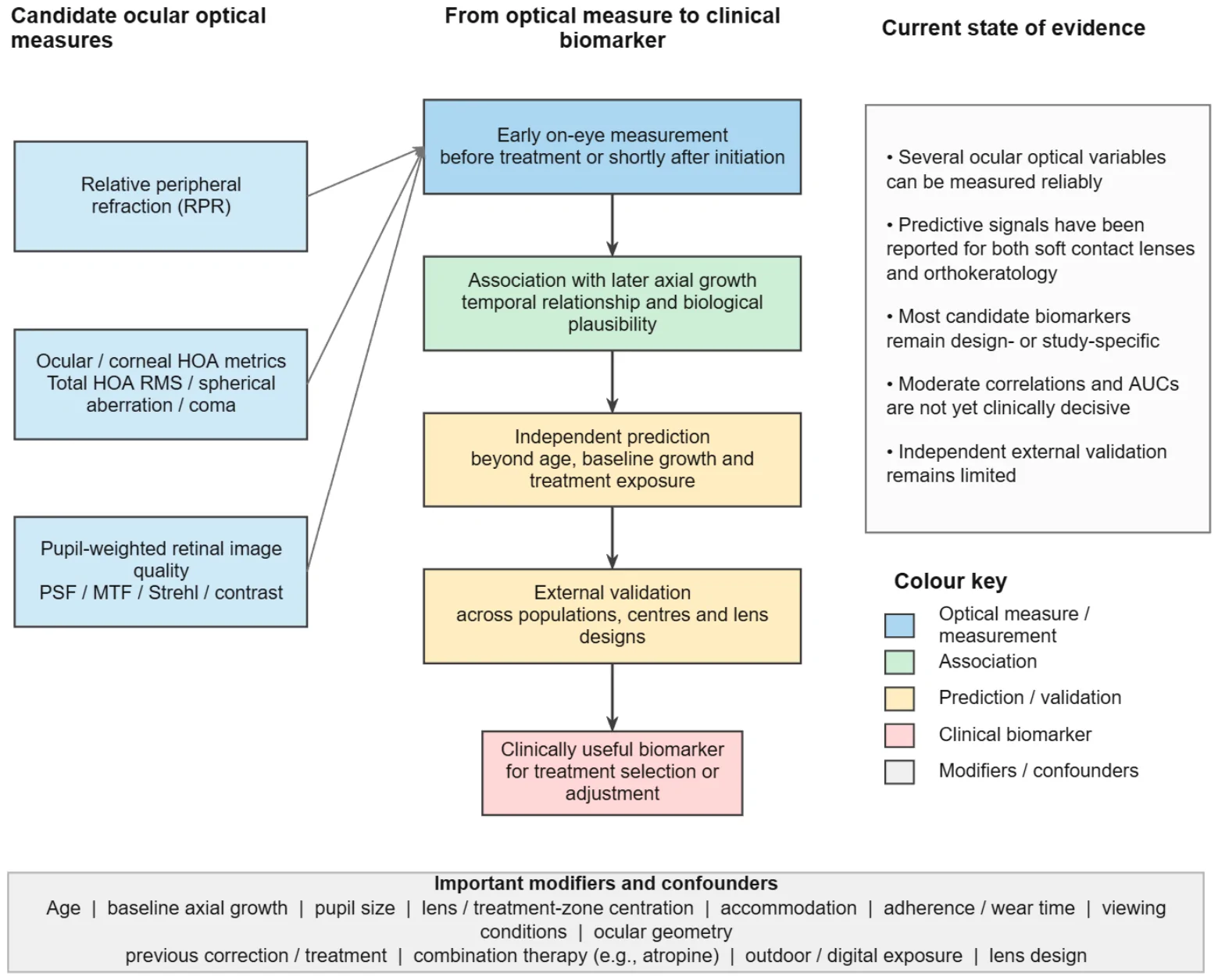
Conceptual pathway for evaluating candidate ocular optical biomarkers of response to contact-lens myopia control. Candidate optical measures progress from reliable early measurement and association with subsequent axial elongation to independent prediction and external validation. Accommodation, pupil size, lens or treatment-zone centration, wear time and viewing conditions are treated as modifiers of optical exposure rather than optical biomarkers. The higher-order aberration candidates refer principally to total HOA root-mean-square, spherical aberration and coma, reflecting the measures for which longitudinal or predictive evidence is currently available.

A broader view of treatment response is supported by studies showing altered central retinal electrophysiology during dual-focus lens wear [34] and by experimental contact lenses designed to modify retinal contrast without adding refractive power [35,36]. These observations challenge the assumption that refractive defocus is the only relevant cue. They do not yet establish ocular optical biomarkers of efficacy: electrophysiological responses are biological measures, while the paediatric contrast-modulation work remains preliminary. Their value lies in suggesting that future prediction may combine a measured optical input with an early biological retinal response rather than relying on optics alone. Figure 1 summarises the proposed pathway from candidate ocular optical measures to a clinically useful biomarker and distinguishes optical measures from modifiers of optical exposure and biological responses.

## 5. Soft Contact Lenses: Emerging Predictive Evidence

### 5.1. Design Strength Predicts Groups Better than Individuals

Soft contact-lens trials provide convincing evidence that optical design influences average treatment efficacy. Early dual-focus and concentric-defocus studies established proof of concept [37,38,39]. The three-year MiSight trial showed substantially less axial elongation with a dual-focus lens than with a same-material single-vision lens [2], while BLINK demonstrated a larger effect with +2.50 D than with +1.50 D centre-distance multifocal optics [3]. More recent EDOF and concentric-defocus trials reinforce the design-specific nature of efficacy [4,40,41,42,43].

These findings describe dose-response at the level of the intervention rather than prediction at the level of the individual child. The same nominal add is modified by accommodation, pupil geometry, centration, the ocular wavefront and the number of hours the lens is actually worn. Wear-time effects have been reported in soft-lens trials, supporting treatment exposure as an important covariate [4,38]. Children wearing the same design nevertheless continue to show a wide range of axial outcomes. BLINK illustrates the distinction particularly well: stronger multifocal optics improved average efficacy, but measured peripheral-defocus variables explained less once treatment assignment was included, and pupil size did not modify the +2.50 D effect [3,11]. A group-level optical dose-response cannot therefore be assumed to define an individual optical threshold.

### 5.2. Relative Peripheral Refraction: Promising, but Not Consistently Independent

The 2025 prospective MiSight study is the clearest soft-lens example in which an early optical change was tested against later growth. In 44 children, three-month changes in total RPR and several wide-field regions were negatively correlated with annual axial elongation; changes in total RPR and the 45-degree field each showed correlations of approximately r = −0.43. Selected RPR changes produced areas under the receiver-operating-characteristic curve of 0.70–0.76 when faster and slower growth groups were compared [9]. These values indicate moderate discrimination rather than a clinically decisive test: correlations of this magnitude account for less than 20% of the observed variance, and the small sample, lack of a parallel control group and absence of an independent validation cohort further limit individual prediction.

The finding is best considered alongside the BLINK analysis. In BLINK, peripheral-defocus associations were no longer convincing after randomised treatment group was included in the model [11]. The two studies are not direct replications: they used different lenses, instruments, retinal sampling and analytical questions. Together, they suggest that RPR may contain predictive information in some settings, but not as a simple universal scalar. Development of an RPR biomarker will require the spatial-summary feature to be selected before analysis and then tested in an independent cohort.

### 5.3. Accommodation Provides an Early, Design-Specific Response Modifier

Accommodation deserves separate consideration because it changes the optical state produced during near viewing and the available soft-lens evidence includes an early, temporally ordered association. Cheng, Xu and Brennan analysed 109 children from a one-year trial of a positive-spherical-aberration myopia-control lens and measured accommodative response after one week of treatment. Within the test-lens group, greater accommodative response was associated with less axial elongation at one year, while no significant relationship was found in the single-vision control group [10]. The finding is therefore potentially useful for prediction, but accommodation itself is a physiological response rather than an ocular optical biomarker.

The finding remains preliminary as a predictor. The analysis was post hoc, the lens relied on positive spherical aberration rather than the geometry of current dual-focus products, and no predictive cut-off was derived or externally tested. A 2025 MiSight study of sustained electronic-device viewing provides physiological support by showing that accommodation and retinal defocus change during prolonged near work [33], although those measurements were not linked to subsequent axial growth. Accommodation is therefore better viewed as a dynamic dose modifier and candidate predictive covariate than as a validated optical biomarker.

### 5.4. Retinal Image-Quality Metrics Remain Biologically Attractive but Unvalidated

Soft lenses are well suited to wavefront and image-quality analysis because the lens optics can be characterised and combined with the ocular wavefront. Existing work shows substantial differences between myopia-control lenses in power profile, point-spread function, modulation transfer and visual performance [7]. Modelling across accommodation provides an even richer description of the retinal image [8]. The clinical efficacy trials, however, have rarely collected these measurements in a form that permits prospective linkage to individual axial response.

The limitation is therefore not a lack of measurable optics, but a lack of longitudinal prediction studies using those measurements. Soft-lens evidence already includes a positive early wide-field RPR signal [9], a temporally ordered accommodation signal [10], a large-trial negative result for simple peripheral defocus and pupil size [11], and increasingly sophisticated measurements of delivered dose and dynamic retinal image quality [6,7,8,33]. The literature is best characterised as emerging and internally mixed rather than uniformly weak. The most useful future models are likely to distinguish the optical predictor itself from variables that modify how that predictor is delivered. The soft contact-lens evidence relevant to this biomarker question is summarised in Table 2.

## 6. Orthokeratology: A More Developed Predictive Literature

### 6.1. Why Orthokeratology Is Informative

Orthokeratology currently provides a larger cluster of studies in which early or treatment-induced optical changes are related prospectively to later axial growth. This difference should not be interpreted as weaker biological evidence for soft contact lenses. The delivery modes are fundamentally different. Soft myopia-control lenses are worn during waking hours and provide their optical profile only while on the eye; orthokeratology lenses are worn during sleep, removed on waking and leave a reshaped corneal surface whose optical state changes during the subsequent waking period [19]. Corneal reshaping also produces relatively large and readily measurable changes in topography, peripheral power and higher-order aberrations, making optical-response relationships easier to study. The underlying efficacy evidence is sufficiently established to support investigation of these predictors [5,20,44,45].

At the same time, orthokeratology illustrates the difficulty of assigning causality to any one optical measure. A smaller treatment zone can alter peripheral refraction, spherical aberration, coma and pupil overlap simultaneously [15,22,23,24,25,30]. When several of these variables move together and each is associated with slower axial elongation, a statistical predictor may emerge without identifying the optical cue at the retina that influences eye growth.

### 6.2. Region-Specific Peripheral Defocus

Peripheral-refraction studies have moved beyond single nasal and temporal measurements. The randomised CONTROL study confirmed that orthokeratology produces myopic relative peripheral refraction, but the relationship with axial growth was not simple enough to support one universal metric [26]. More recent wide-field work has reported stronger associations in selected retinal regions. Prospective studies have linked greater myopic shifts in particular annuli or retinal sectors with less axial elongation, and a 2026 wide-angle study identified selected peripheral astigmatic components measured at six months as predictors of later treatment response [12,13,14].

These studies strengthen the case for spatial peripheral optics while also making clear that the candidate is more complex than a single eccentric refraction value. A clinically useful biomarker may need to describe the shape, region or distribution of peripheral defocus and astigmatism. Replication is particularly important because instruments differ in field of view, sampling density and regional definitions.

### 6.3. Total HOA RMS, Spherical Aberration and Coma as Predictive Candidates

Within orthokeratology, the clearest longitudinal and predictive evidence concerns total HOA RMS, spherical aberration and coma rather than HOAs as an undifferentiated group. Earlier longitudinal work found higher total ocular HOA RMS and spherical-aberration RMS, and more positive primary spherical aberration Z4^0, to be associated with slower axial elongation [16]. The 2025 prospective cohort subsequently showed that change in total anterior corneal HOA RMS and change in horizontal coma contributed independently to prediction of later axial elongation [17].

Among these metrics, change in total anterior corneal HOA RMS provided the strongest single-variable discrimination, with an AUC of 0.803 and a proposed cut-off of 0.834 micrometres; change in horizontal coma produced an AUC of 0.756 [17]. These values remain context-dependent because HOA magnitude varies with pupil diameter, Zernike convention, corneal versus total-eye measurement and timing relative to orthokeratology lens removal [15,17,19]. Total HOA RMS is therefore the leading predictive candidate in the current orthokeratology literature, while primary spherical aberration and horizontal coma provide component-specific evidence with different strengths. Other HOA terms should not be assumed to have equivalent predictive value without direct longitudinal validation.

### 6.4. Treatment-Zone Geometry Remains an Important Surrogate

The VOLTZ trial and subsequent smaller-zone studies provide unusually direct evidence that changing orthokeratology geometry changes efficacy [22,23,24,25,27]. Observational evidence also links treatment-zone decentration with axial elongation [30]. Treatment-zone size and position are therefore useful engineering surrogates and may help guide lens design before the causal optical cue at the retina is fully understood. For biomarker development, however, geometry is most informative when measured alongside the RPR, HOA and pupil-weighted image-quality changes that it produces. The orthokeratology evidence most relevant to optical prediction is summarised in Table 3.

## 7. Current Predictive Value and Evidence Hierarchy Across Contact-Lens Modalities

### 7.1. Current Candidate Signals

The current literature contains several temporally ordered signals, but their evidential status differs. In orthokeratology, change in total anterior corneal HOA RMS has shown the strongest single-study discrimination, with an AUC of 0.803 [17]. Region-specific RPR has prospective predictive evidence in both orthokeratology and soft multifocal lens wear, including AUCs of 0.70–0.76 for selected early RPR changes in the MiSight cohort [9,12,13,14]. In soft lenses, accommodative response measured after one week has also been associated with one-year axial elongation within a positive-spherical-aberration design [10], although accommodation is better regarded as a modifier of optical exposure than as an optical biomarker.

No single candidate performs consistently across contact-lens modalities. The proposed 0.834 micrometre threshold refers specifically to change in total anterior corneal HOA RMS and was derived in a single-centre orthokeratology cohort [17]. RPR can be measured in both soft-lens and orthokeratology treatments, but BLINK indicates that simple peripheral defocus may not independently explain the soft-lens treatment effect [11]. Accommodation currently has temporally ordered evidence for one soft-lens design [10], treatment-zone geometry remains an engineering surrogate, and pupil-weighted retinal image quality still lacks prospective outcome validation. The available pattern therefore favours modality-specific or multivariable signatures rather than a universal scalar biomarker.

### 7.2. Statistical Association Is Not Clinical Prediction

A statistically significant association does not establish clinically meaningful prediction. The MiSight RPR correlations of approximately r = −0.43 account for less than one fifth of the observed variance and arose from a small, uncontrolled cohort in which several spatial summaries were examined [9]. The accommodative finding was post hoc and design-specific [10], while the 0.803 HOA AUC and its proposed threshold were estimated in the same single-centre discovery cohort [17]. These findings identify plausible predictive signals, but they do not show that an individual child can be classified reliably in another clinic, population, instrument or lens design.

Clinical prediction requires more than correlation or a favourable AUC. A credible biomarker should be repeatable, well calibrated, incrementally informative beyond age, previous progression and verified treatment exposure, externally validated, and linked to a management decision that improves outcomes. Small samples, multiple correlated candidate metrics, exploratory regional selection, post hoc analyses and thresholds derived and assessed in the same dataset can all inflate apparent performance [9,10,17,29]. Within the literature reviewed, no ocular optical candidate has yet demonstrated independent external validation or shown that using the marker to select or adjust treatment improves axial outcomes. The evidence therefore supports candidate predictors rather than clinically actionable biomarkers.

Table 4 summarises the current validation level of the principal optical candidates and delivery modifiers.

## 8. Why Has a Clinically Validated Biomarker Not Yet Emerged?

### 8.1. Optical Variables Are Coupled

Most myopia-control lenses alter several optical properties at the same time. Increasing add power changes through-focus image quality [3,6,7,8]; reducing an orthokeratology treatment zone changes peripheral refraction and higher-order aberrations [15,22,23,24,25]; and treatment-zone or lens decentration changes the spatial relationship between the optical profile and the pupil [30,31]. Statistical models can separate correlated variables only imperfectly, particularly in small cohorts. A variable may therefore be useful for prediction without being the causal signal, while a causal cue may be distributed across several correlated measurements.

### 8.2. The Measurements Are Often Static but Treatment Is Dynamic

The retinal image changes with pupil diameter, accommodation, gaze, illumination and lens position. Modern soft lenses can be systematically decentred relative to the pupil centre [31], while accommodation alters retinal vergence during near work; the one-week accommodation result [10] and the 60-min dual-focus near-viewing study [33] show that these dynamic effects are measurable. Orthokeratology adds a time-of-day component because the post-treatment corneal optical state changes after lens removal [19]. Most biomarker studies still rely on a single viewing distance, pupil condition and visit, so a repeatable snapshot may nevertheless fail to represent habitual optical exposure at the retina.

### 8.3. Response Is Not Defined Consistently

Definitions of a treatment “responder” vary considerably between studies. Outcomes include absolute axial elongation, percentage slowing relative to a control, age-adjusted growth and fixed thresholds such as 0.10 or 0.11 mm/year. Because normal ocular growth changes with age [46], the biological meaning of a fixed threshold is not the same in an eight-year-old and a fifteen-year-old. Biomarker validation will therefore require a pre-specified outcome that accounts for age and, where possible, expected untreated growth rather than relying solely on an arbitrary cut-off.

### 8.4. Clinical and Behavioural Factors Can Dominate

Optical quality alone cannot explain response if the intended treatment is not consistently delivered. Wearing time influenced outcomes in early soft-lens studies [4,38], and a small predictive analysis of MiSight users found time outdoors to be the only independent factor among a broad set of baseline clinical, binocular and wavefront variables [29]. BLINK also found no evidence that pupil size modified the high-add multifocal treatment effect [11]. Predictive studies should therefore record relevant non-optical covariates rather than assume that an optical marker acts in isolation. Depending on the study population, these may include age, baseline progression, outdoor or digital-device exposure, previous correction or treatment history, adherence and relevant ocular or systemic health. These factors were not reviewed comprehensively because they fall outside the primary optical-biomarker question. Combination therapy introduces an additional optical consideration: in the Bifocal and Atropine in Myopia study, adding 0.01% atropine to +2.50 D multifocal soft lenses increased photopic pupil diameter relative to multifocal lens wear alone, while providing no clear additional myopia-control benefit [47]. Pharmacological pupil enlargement can therefore change the fraction of a multifocal power profile sampled by the pupil even when the lens design itself is unchanged.

### 8.5. Engineering Evidence on Patient-Specific On-Eye Optics

A complementary body of engineering work supports the premise that nominal lens specifications do not uniquely determine the optical state produced on eye. Computational and experimental studies have shown that soft-lens fitting can alter effective power, with the magnitude influenced by base curve and diameter, hydrogel material properties, peripheral stabilisation design, corneal shape and compressive behaviour [48,49,50,51]. These studies were not designed to predict axial elongation and therefore should not be interpreted as biomarker-validation evidence. Their relevance is methodological: they demonstrate that individual lens–eye interaction can modify the optical exposure generated by a nominally identical lens and therefore support measuring, rather than assuming, the on-eye optical state.

Related orthokeratology work has linked base- and reverse-curve design parameters to central and annular refractive-power changes [52], used patient-specific finite-element stress–strain features to forecast corneal refractive-power change [53], and applied machine learning to predict the magnitude and direction of treatment-zone decentration associated with induced HOAs and astigmatism [54]. These models do not yet predict myopia-control response, but they provide a useful engineering bridge between lens design, patient-specific fit and measurable optical consequences. Future biomarker studies could test whether model-derived estimates add predictive value to directly measured RPR, wavefront and retinal image-quality metrics.

## 9. A Practical Validation and Future-Research Pathway

Future work should be designed as prediction research rather than as a search for further associations. A prospective, preregistered, multicentre design with a justified sample size, a small set of candidate metrics and an analysis plan fixed in advance would reduce selective reporting and overfitting. The optical core should remain modality-aware. In orthokeratology, reasonable candidates include pupil-standardised total HOA RMS, primary spherical aberration, horizontal coma, a spatial RPR descriptor and a pupil-weighted image-quality metric [12,13,14,15,16,17]. In soft lenses, wide-field RPR, pupil-standardised ocular-wavefront measures and pupil-weighted retinal image quality currently have the strongest optical rationale [6,7,8,9,32]. On-eye centration, pupil size, accommodation and verified wear time should be recorded separately as modifiers of optical exposure [10,11,31,33].

Measurement timing should match the candidate under study. Accommodation can be assessed within the first week as a dynamic modifier, whereas RPR and orthokeratology wavefront metrics can be repeated after the early optical state has stabilised, commonly within one to three months [9,10,17]. Axial length should then be followed continuously for at least 12 months and preferably 24 months, with age-adjusted or normative context rather than an arbitrary responder threshold as the principal outcome. Prediction models should compare a clinical reference model with an optical-augmented model and include age, baseline AL/SER, previous progression, treatment history, site or population, relevant behavioural exposure and verified wear as pre-specified covariates.

Model development should limit the number of predictors, address missing data explicitly and use bootstrap resampling or nested cross-validation to estimate optimism. Reporting should include calibration-in-the-large and calibration slope, discrimination with confidence intervals, measurement repeatability and decision-curve or net-benefit analysis; statistical significance and AUC alone are insufficient. The model and any thresholds should then be locked before independent external validation across centres, instruments, populations and lens designs. Where direct on-eye measurement is impractical, patient-specific modelling may provide a complementary estimate of the optical state, but model-derived metrics still require prospective validation against axial outcomes [48,49,50,51,52,53,54].

Only after reproducible external validation should actionability be tested. A prospective clinical study should compare biomarker-guided management with standard age-aware axial monitoring and assess axial outcomes, unnecessary treatment switching, adverse consequences, patient burden and cost. Table 5 consolidates these minimum design, validation, reporting and clinical-utility requirements.

## 10. Clinical Interpretation and Implications

### 10.1. Current Supportive Role

At present, age-aware axial-length monitoring remains the primary method for judging treatment response [46]. Ocular optical measurements may serve a supportive role by documenting whether the intended on-eye optical state is being achieved and by helping to troubleshoot poor centration, an unexpectedly large orthokeratology treatment zone, weak pupil-zone overlap or an atypical RPR or wavefront pattern [22,23,24,25,30,31]. Accommodation and verified wear time may also explain why the intended optical exposure is not sustained during habitual viewing [4,10,33,38]. Used in this way, optical measurements can inform fitting, adherence discussions and monitoring without being presented as validated predictors of an individual child’s response.

### 10.2. Decisions Not Currently Supported

No current ocular optical measure should be used alone to choose the initial lens, classify a child as a responder or non-responder, switch treatment modality, add combination therapy or reduce follow-up. The MiSight wide-field RPR AUCs of 0.70–0.76 are insufficient for sole clinical decisions [9]; the orthokeratology cut-off of approximately 0.834 micrometres applies specifically to change in total anterior corneal HOA RMS and remains unvalidated outside its discovery setting [17]; and BLINK does not support pupil size as a criterion for selecting high-add multifocal treatment [11]. Predictive accuracy alone would still be insufficient: clinical use requires calibrated individual estimates, a defensible decision threshold and evidence that acting on the result improves outcomes compared with standard care.

### 10.3. Potential Future Applications

If validated, ocular optical biomarkers could support personalised lens selection or design, earlier recognition of inadequate optical exposure, fitting optimisation and risk-stratified follow-up. Their most plausible use is likely to be within modality-specific multivariable models that combine optical measurements with age, prior progression, treatment history and verified exposure rather than as stand-alone thresholds. These applications should remain investigational until external validation and a prospective comparison of biomarker-guided management with standard age-aware axial monitoring demonstrate clinical net benefit.

## 11. Limitations of the Evidence and of This Review

The evidence base remains heterogeneous, and the apparently different soft-lens findings illustrate why direct comparison is difficult. The positive MiSight RPR study was a small, non-controlled prospective cohort [9], while the accommodation finding arose from a post hoc analysis of a positive-spherical-aberration lens design [10]. BLINK was much larger and randomised, but its peripheral-defocus analysis addressed whether defocus independently explained the treatment-group effect rather than whether a pre-specified early change predicted later response within a single design [11]. Orthokeratology studies are frequently single-centre and concentrated in East Asian populations, with optical metrics measured using different instruments, pupil diameters and post-waking intervals. Peripheral-refraction instruments also differ in sampling aperture, which may be important when power varies rapidly across a contact-lens treatment zone [28]. These differences limit transportability of both positive and negative findings.

Prediction-specific methodological limitations also constrain interpretation. Several positive findings come from small or single-centre cohorts, post hoc analyses, exploratory selection of retinal regions or correlated optical variables, and thresholds derived and evaluated in the same dataset [9,10,17,29]. These practices can produce optimistic estimates of discrimination. AUC values without calibration, confidence intervals, comparison with a clinical reference model, transparent handling of missing data and independent external validation cannot establish reliable individual prediction or clinical utility. Negative findings are also difficult to transport when instruments, pupil conditions, spatial sampling and statistical questions differ between studies.

The review is narrative rather than systematic and does not include formal risk-of-bias grading or meta-analysis. Its purpose is to clarify the distinction between optical association and prediction and to assess how far current evidence has progressed towards a clinically useful optical biomarker. Spectacle-based myopia-control systems were excluded deliberately, and non-optical determinants such as environmental exposure, previous correction history and general biological health were treated as potential covariates rather than reviewed comprehensively. The conclusions should therefore be read as a critical synthesis rather than a validated ranking of candidates.

## 12. Conclusions

Current literature supports candidate ocular optical predictors rather than clinically validated biomarkers. Region-specific RPR and treatment-induced total anterior corneal HOA RMS provide the most promising preliminary discrimination, particularly in orthokeratology, but the evidence is based largely on small, single-centre or design-specific studies and lacks independent external validation, calibration and demonstrated incremental value beyond established clinical factors [9,12,13,14,15,16,17]. Accommodation, pupil size, centration and wear time should be treated as modifiers of optical exposure rather than stand-alone biomarkers [10,11,31,33]. No current measure should determine lens selection, treatment switching or non-responder classification; optical measurements may instead support troubleshooting alongside age-aware axial monitoring. Future research should be prospective, preregistered and multicentre, use standardised optical protocols and continuous age-aware outcomes, compare clinical-only with optical-augmented models, report calibration, discrimination and decision-curve utility, and complete independent external validation. Biomarker-guided management should then be tested against standard care. Modality-specific multivariable signatures remain more plausible than a single universal biomarker.

## Figures and Tables

**Table 1:** Criteria used in this review to judge candidate ocular optical biomarkers.

Criterion	What Would Support Biomarker Status	Common Weakness in the Current Literature	Why It Matters Clinically
Timing	Measured at baseline or within the first weeks/months of treatment	Optics and axial elongation are often measured at the same late visit	Prediction must precede the outcome
Repeatability	Stable measurement under defined pupil and accommodative conditions	Different devices, pupil diameters and eccentricities are used	A threshold is not portable if the metric changes with the protocol
Independent prediction	Adds information beyond age, baseline AL/SER and prior progression	Small cohorts and many correlated optical variables	A biomarker should improve prediction, not simply restate known risk
Generalisability	Replicated in an external cohort and ideally another lens design	Most positive studies are single-centre and design-specific	Clinical use requires performance beyond the discovery dataset
Actionability	Suggests a change in fit, design or treatment when the signal is inadequate	Validated cut-offs are largely absent	A marker is most useful when it can change management

**Table 2:** Soft contact-lens evidence viewed through the biomarker question.

Evidence	What It Shows	Biomarker Relevance	Current Limitation
MiSight 3-year RCT [2]	Strong average reduction in axial elongation	Establishes a robust response phenotype for a defined design	Does not identify an early on-eye optical predictor of individual response
BLINK efficacy trial [3]	+2.50 D add outperformed +1.50 D at group level	Supports optical-strength dependence	Nominal add is a treatment parameter, not an individual ocular optical biomarker
BLINK peripheral-defocus analysis [11]	Peripheral-defocus associations weakened after treatment group was included; pupil size did not modify the high-add effect	Important negative test of simple defocus and pupil-size hypotheses	Does not exclude more complex or spatially integrated optical inputs at the retina
MiSight wide-field RPR cohort [9]	Three-month RPR changes correlated with later AL growth; selected AUCs 0.70–0.76	Direct early-response biomarker-development evidence	44 children, non-controlled, multiple regional metrics, no external validation
Positive spherical-aberration accommodation analysis [10]	One-week accommodative response associated with one-year AL change within the test-lens group	Temporally ordered, design-specific response modifier with predictive potential	Post hoc analysis; accommodation is physiological rather than an optical biomarker; no validated cut-off or independent replication
Delivered-dose and dynamic-defocus studies [6,33]	Pupil, accommodation and sustained near viewing alter the optical state produced at the retina	Shows why static-labelled optics differ from real exposure and why wear time and accommodation should be modelled separately	Mechanistic/dynamic measurements not linked prospectively to later AL growth
Image-quality characterisation/model [7,8]	Quantifies pupil-weighted and through-focus optical differences	Provides integrated candidate metrics for future prediction	Mainly optical or model-based rather than longitudinal predictive data

**Table 3:** Orthokeratology studies most relevant to predictive ocular optics.

Candidate Measure	Representative Evidence	Signal Related to Axial Response	Biomarker Status
RPR/regional peripheral defocus	[12,13,14,26]	Several prospective studies link more myopic regional shifts or defocus-ring features with less elongation	Promising; spatial definitions and predictive regions are not yet consistent.
Total HOA RMS/primary spherical aberration/horizontal coma	[16,17]	Higher total HOA RMS and spherical-aberration metrics have been associated with slower elongation; Δtotal anterior corneal HOA RMS showed AUC 0.803 and Δhorizontal coma AUC 0.756 [16,17]	Total HOA RMS is the leading predictive candidate within orthokeratology; component-specific evidence is strongest for primary spherical aberration and horizontal coma. External validation is required.
Treatment-zone diameter/BOZD	[22,24,25]	Smaller zones generally associated with less axial elongation	Strong design surrogate, but not itself an ocular optical output.
Treatment-zone decentration/pupil overlap	[22]	Changes the spatial position of power and aberrations across the pupil	Plausible delivery modifier; optimal direction and magnitude remain uncertain.
Integrated image-quality metric	[15]	Could combine defocus and aberration into one retinal representation	Biologically attractive but little prospective prediction evidence in treated children.

**Table 4:** Current validation status of candidate optical measures and delivery modifiers in 2026.

Candidate	Current Validation Level	Main Reason for Interest	What Is Still Missing
Treatment-induced total HOA RMS/selected components	Preliminary internal prediction within orthokeratology; single-centre	Prospective discrimination for Δtotal anterior corneal HOA RMS (AUC 0.803); horizontal coma also predictive in one cohort [17]	Independent external validation, standardised pupil/wavefront protocol, calibration, incremental value beyond clinical predictors, and evidence beyond orthokeratology
Region-specific/wide-field RPR	Preliminary prediction; mixed across designs and spatial definitions	Prospective predictive signals in orthokeratology and MiSight; MiSight AUCs 0.70–0.76 [9,12,13,14]	A pre-specified spatial metric, replication of the MiSight signal, reconciliation with BLINK [11], calibration and independent external validation
Accommodation/accommodative response	Post hoc association; design-specific; not a validated optical biomarker	One-week response associated with one-year AL growth in a positive spherical aberration lens [10]	Prospective replication, a pre-specified model, incremental value beyond clinical factors and independent external validation
Treatment-zone geometry/pupil overlap	Strong intervention/design evidence; not biomarker prediction	Randomised evidence that geometry changes orthokeratology efficacy [22,23,24,25]	Direct evidence that a measured optical consequence predicts outcome and that acting on it improves management
Pupil-weighted retinal image quality	Mechanistic and measurement evidence; no prospective prediction	Integrates defocus, aberrations and pupil weighting [7,8]	Prospective linkage to later AL growth, calibration, external validation and clinically useful thresholds
Pupil size alone	Negative or weak as a stand-alone predictor	Easy to measure, but BLINK found no modification of the high-add effect [11]	Evidence of incremental value within a pre-specified multivariable signature and independent external validation

**Table 5:** Minimum design, validation and reporting requirements for a study intended to establish an ocular optical biomarker of myopia-control response.

Study Element	Recommended Approach	Rationale
Study design	Prospective, preregistered, multicentre cohort with a justified sample size; development and validation populations defined in advance.	Reduces selective reporting and overfitting and supports transportability.
Timing	Baseline plus an early post-treatment visit matched to the candidate (about 1 week for dynamic accommodation; 1–3 months for RPR/HOAs).	Allows the candidate to precede the main axial outcome.
Optical core	Pre-specified, modality-specific metrics: soft lenses—wide-field RPR, pupil-standardised wavefront measures and pupil-weighted retinal image quality; orthokeratology—RPR, pupil-standardised total HOA RMS, primary spherical aberration, horizontal coma and retinal image quality.	Keeps the optical predictor distinct from physiological and fitting modifiers.
Dynamic state	Defined distance and near conditions plus accommodation, pupil size, centration and verified wear for soft lenses; standardised pupil and time after waking for orthokeratology.	Captures the conditions under which optical exposure occurs.
Clinical covariates	Age, baseline AL/SER, prior progression, treatment history, site/population, outdoor and digital exposure, relevant health factors and verified wear.	Tests whether the optical measure adds value beyond established clinical, behavioural and treatment-exposure predictors.
Outcome	Continuous absolute axial elongation with age-adjusted or normative context over 12–24 months; fixed responder thresholds used only as secondary analyses.	Avoids unstable percentage efficacy and arbitrary responder labels.
Model development	Compare clinical-only and clinical-plus-optical models; limit predictor count; address missing data; use bootstrap or nested cross-validation and report optimism-corrected performance.	Tests incremental predictive value while reducing overfitting.
External validation	Lock the model and thresholds before testing in independent centres, instruments, populations and lens designs.	Determines whether performance is transportable beyond the discovery dataset.
Reporting	Calibration-in-the-large and slope, discrimination with confidence intervals, decision-curve/net benefit, measurement repeatability and missing-data handling.	AUC or statistical significance alone does not establish reliable individual prediction or clinical usefulness.
Clinical utility	After external validation, compare biomarker-guided management with standard age-aware axial monitoring in a prospective trial.	Determines whether acting on the biomarker improves outcomes and justifies burden and cost.

## Data Availability

No new data were created or analysed in this study. Data sharing is not applicable to this article.

## Abbreviations

The following abbreviations are used in this manuscript:

Abbreviation	Definition
AL	axial length
AUC	area under the receiver-operating-characteristic curve
BOZD	back optic-zone diameter
EDOF	extended depth of focus
HOA	higher-order aberration
MTF	modulation transfer function
PSF	point-spread function
RCT	randomised controlled trial
RPR	relative peripheral refraction
SER	spherical equivalent refraction
